# Properties and Application of Edible Modified Bacterial Cellulose Film Based Sago Liquid Waste as Food Packaging

**DOI:** 10.3390/polym13203570

**Published:** 2021-10-16

**Authors:** Nur Arfa Yanti, Sitti Wirdhana Ahmad, La Ode Ahmad Nur Ramadhan, Jamili, Muzuni, Taufik Walhidayah, Jendri Mamangkey

**Affiliations:** 1 Department of Biology, Faculty of Mathematics and Natural Science, Halu Oleo University, Kendari 93232, Southeast Sulawesi, Indonesia; wirdhanaaxtalora@yahoo.com (S.W.A.); jamili66@yahoo.com (J.); muzuni71@yahoo.co.id (M.); twhidayatwahid@gmail.com (T.W.); 2 Department of Chemistry, Faculty of Mathematics and Natural Science, Halu Oleo University, Kendari 93232, Southeast Sulawesi, Indonesia; ramadhan305@gmail.com; 3 Department of Biology Education, Faculty of Education and Teacher Training, Universitas Kristen Indonesia, Jakarta Timur 13630, Jakarta, Indonesia; jendrimamangkeybiology@gmail.com

**Keywords:** bacterial cellulose, edible film, food packaging, sago liquid waste

## Abstract

Bacterial cellulose (BC) based on sago liquid waste has been developed to be used as food packaging. This study investigated the physicochemical and mechanical properties of modified BC film and its application as food packaging. The modified BC film performed carboxymethyl cellulose (CMC) as a stabilizer and glycerol as a plasticizer. Films were prepared by casting technique using BC as the primary material and composites with various concentrations of CMC and glycerol (0.5%, 1%, and 1.5%, *v*/*v*). BC film was applied as the packaging of meat sausage, and the quality of meat sausage was measured based on weight loss, moisture content, pH, protein content, and total microbial count. The addition of CMC and glycerol influences the physical and mechanical properties of BC composites film. The best mechanical properties of edible BC film were collected by adding 1% CMC and 1% glycerol with a tensile strength of 17.47 MPa, elongation at a break of 25.60%, and Young’s modulus of 6.54 GPa. FTIR analysis showed the characteristic bands of BC, and the addition of CMC and glycerol slightly changed the FTIR spectrum of the composites. The utilization of modified BC-based sago liquid waste film as the packaging of meat sausage could maintain sausage quality during 6 days of storage at room temperature. Therefore, edible BC film has the potential to be used as food packaging.

## 1. Introduction

Plastic has dominated the global market share of packaging material in the last two decades, replacing cans and glass packaging. Food packaging using plastic has become the major contributor to global plastic pollution up to 90%, especially in middle- and low-income countries [1], since its lack of waste management systems. Furthermore, the monomer of plastic packaging and other small molecules of plastic could introduce some carcinogenic chemical components, even poisoning the body through toxicity [2]. Therefore, environmentally friendly and safe plastic is needed regarding the health and environmental issue.

The biological approach was used as an alternative method to mitigate the use of synthetic plastics. The recent biodegradable plastics (BPs) development has been developed from bacterial biomass, which is becoming an interesting topic to be studied [3,4,5], mainly to be applied as scaffolding materials, bone tissue engineering, nerve regeneration, blood vessel replacement [4], food industry [4,5], and burn wound dressing [4]. BC is one of the promising biomaterials which can be developed as a food packaging plastic material [6] that is produced by acetic acid bacteria through the fermentation of high carbohydrate-containing substrates such as agricultural and industrial waste [7,8,9]. The BC production is mainly facilitated in the laboratory by non-pathogenic bacteria, such as *Acetobacter xylinum* [7,8,9,10] and *Gluconacetobacter xylinum* [11,12].

Plastic food packaging products derived from BC has become the focus of this study due to its superior biodegradability properties in the nature. The synthetic plastics currently and widely used in the food and non-food industries can be replaced by BC because the cellulose components are free of lignin, have high mechanical properties, and do not damage the environment (biodegradable) [13].

Given this problem, the abundant waste of polysaccharides in Southeast Sulawesi, Indonesia, or hence the popular sago liquid waste by-products produced from the sago starch processing industry were disposed of in the surrounding environment. In order to mitigate the environmental issue due to the use of synthetic plastics, sago liquid waste was treated to produce BC [9,10]. The properties of BC from sago liquid waste were supportive to be applied as plastic [6] so that BC has the potential to be made into an edible film for food packaging. Therefore, the purpose of this study was to evaluate the properties and applications of edible films from BC by utilizing sago liquid waste substrate as food packaging plastic.

## 2. Materials and Methods

### 2.1. Materials

The main ingredient for making BC is sago liquid waste from the sago processing industry in Southeast Sulawesi, Indonesia. *Acetobacter xylinum* strain LKN6 used in this study for BC production is a local strain isolated from pineapple skin waste [10] and prepared in the form of liquid inoculum (starter) using coconut water substrate. The making of edible films used the treatment of adding concentrations (%, *v*/*v*) of carboxy-methyl cellulose (CMC) and glycerol from 0, 0.5, 1, and 1.5. All chemicals were purchased from Merck Chemical Co. (USA).

### 2.2. *Production of Bacterial Cellulose*

BC was produced using sago liquid waste as a substrate following the research conducted by Yanti et al. [10]. The production media were incubated at room temperature without agitation for 14 days. After incubation, the BC layer formed on the top of the fermentation medium was removed and washed using clean water at least three times to ensure the cleanliness and then used to make the edible film.

### 2.3. Production of Edible Film

BC gel was washed using 1% NaOH solution to remove the non-cellulose component until the pH became neutral. BC was added water in ratio BC and water 1: 4 and blended until it became slurry which was then used as the essential ingredient for making the edible film. The treatment was then carried out by adding the BC slurry with additive compounds, namely CMC and glycerol, with the composition according to Table 1. The BC slurry was stirred until homogeneous and allowed to stand for 5 min; after that, the degassing process was carried out by heating it using a hotplate at 150 °C for 1 h. Furthermore, the dough was printed through the casting method and dried in a 40 °C temperature blower oven for one day. The edible film was produced and analyzed based on its physical-mechanical characteristics. The additive compound composition was added to the BC slurry and the treatment was optimized, as listed in Table 1.

### 2.4. Physical Properties

#### 2.4.1. Film Thickness

The film thickness was determined using a manual micrometer (Mitutoyo, Japan) with an accuracy of 0.001 mm. The thickness value was measured at 5 different positions and averaged.

#### 2.4.2. Water Solubility (WS)

The WS of the films was measured according to the method described by Wang et al. [14]. Briefly, samples were cut to size (4 cm × 4 cm) in triplicate and dried at 105 °C for 24 h to determine initial dry weight. The dried films were then immersed in 50 mL of distilled water and stirred at 100 rpm for 24 h at room temperature. After 24 h, any remaining films were taken out and dried at 105 °C for 24 h to obtain the final dry weight. Water solubility samples (%) was calculated through the following equation:

$$\mathrm{WS}\;\left(\%\right)=\frac{\mathrm{initial}\;\mathrm{dry}\;\mathrm{weight}-\mathrm{final}\;\mathrm{dry}\;\mathrm{weight}}{\mathrm{initial}\;\mathrm{dry}\;\mathrm{weight}}\times 100$$

#### 2.4.3. Moisture Content (MC)

The film samples MC was uniformly cut to size (2 cm × 2 cm) in triplicate and dried in an oven at 105 °C for 24 h. After drying, the dry weight of the film was recorded, and the MC was calculated using the following equation [14]:

$$\mathrm{MC}\;\left(\%\right)=\frac{\mathrm{initial}\;\mathrm{sample}\;\mathrm{weight}-\mathrm{dry}\;\mathrm{sample}\;\mathrm{weight}\;}{\mathrm{initial}\;\mathrm{sample}\;\mathrm{weight}}\times 100$$

### 2.5. *Mechanical* *Properties Measurement*

The tensile strength (T) and elongation at break (E) of the films were measured using a universal testing machine (Admet, Norwood, MA, USA), according to the ASTM D638-14 method [15]. Meanwhile, Young’s modulus (*Y*) represents the slope of the linear portion of the tension–strain curve. Data analysis represented the average of 3 replicates for each sample.

### 2.6. *Fourier* *Transformed Infrared (FT-IR) Spectroscopy*

At room temperature, the edible film samples were recorded using FTIR Spectra 2000 (Perkin Elmer, Waltham, MA, US). The spectra were recorded between a wavelength of 500–4000 cm^−^^1^.

### 2.7. *Scanning* *Electron Microscopy (SEM)*

The surface morphology of the edible film was studied by Scanning Electron Microscopy (Tescan Vega III Easyprobe, Brno, Czech Republic). The edible film samples were coated with gold and examined at an accelerated voltage of 5 kV and magnification of 20 k.

### 2.8. *Application* *of Edible BC Film*

Application of edible BC film as food packaging was tested to the meat sausage by the wrapping method. The type of packaging was used in this stage, namely native BC film (without the addition of CMC and glycerol/EF_0-0_) and modified BC film (BC with the addition of 1% (*w/v*) CMC and 1% (*v/v*) glycerol/EF_1-1_), and synthetic plastic (LDPE plastic packaging) was used as the control. After the packaging, the sausage was stored at ambient temperature for 7 days. The weight loss, pH value, total microbial count, moisture content, and protein content were measured to observe the quality of the sausage. The weight loss of sausage was measured using the gravimetric method. The sausage pH was determined using a pH meter that was previously calibrated using a pH 4 and pH 7 solution. The sausage pH was measured by inserting a pH meter into a beaker glass containing a sausage solution and waiting until the pH was constant. The total microbial was counted by the plate count method using plate count agar (PCA) media. The moisture and protein content were analyzed according to the methodology proposed by AOAC [16].

## 3. Results and Discussions

### 3.1. Product of Edible Bacterial Cellulose Film Based Sago Liquid Waste

**Figure 1:**
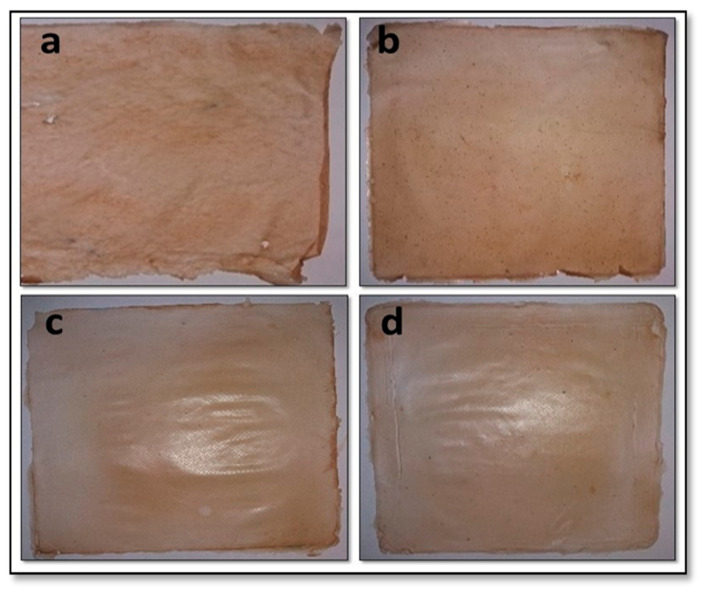
The edible film based BC from sago liquid waste. (**a**) EF0-0 (without CMC and glycerol), (**b**) EF1-1 (CMC 0.5%; glycerol 0.5%), (**c**) EF2-2 (CMC 1%; glycerol 1%), (**d**) EF3-3 (CMC 1.5%; glycerol 1.5%).

Figure 1 shows that the films were brown with an opaque appearance. The brown color of the film was influenced by the color of BC produced from sago liquid waste which is light brown. Yanti et al. [10] reported that the BC pellicle, which was produced using sago liquid waste as substrate, was brownish. Modified BC films with the addition of higher CMC and glycerol faded the brown color of the film with a more transparent appearance (Figure 1). In addition, no air bubbles or holes were ascertained throughout the films since all bubbles were removed by degassing techniques before casting the films. These air bubbles were a concern for film production because they could reduce the tensile strength and other film properties [17]. Control film (EF_0-0_) was made from BC without adding CMC and glycerol, which appeared to be more brittle and easy to tear (Figure 1a) than films with added CMC and glycerol (Figure 1b–d). The results indicated that the addition of CMC and glycerol in BC affected the strength of the film. Laith and Al-Hashimi [18] reported that the presence of CMC and glycerol in edible films could increase the film’s strength.

### 3.2. Physical Properties of Edible Film

Physical properties of the film were measured include thickness, water solubility, (WS), and moisture content (MC). The physical properties of the edible film are shown in Table 2. 

The thickness of the edible film was directly proportional to the increasing CMC and glycerol concentration. The thickness of films increased from 0.045 mm to 0.083 mm with increasing CMC and glycerol concentration (Table 2). These results indicated that adding CMC and glycerol in the BC film can increase the thickness of films. These results are similar to reports of the thickness of edible film-based *Psyllium* seed [19], edible film modified starch/CMC [20], and edible sago film [21]. CMC can form complex solutions to increase viscosity to fill the film’s voids and increase the thickness of film [20]. In addition, the addition of glycerol also increases the total solids in the solution so that the polymer matrix increases, which consequently increases the thickness of edible film [22]. Thickness is an essential factor in determining film clarity, water permeability, and mechanical properties, which increases the film’s ability to improve mechanical food integrity [23].

The water solubility can generally estimate the water resistance of the films. The water solubility (WS) of edible BC modified film was the opposite of the moisture content (MC) of the film (Table 2). The WS of the films decreased from 43.92 to 32.02% by increasing CMC and glycerol concentration, whereas the MC increased from 19.30% to 33.72% (Table 2). These results indicated that CMC and glycerol addition significantly affected solubility in water and moisture content. A similar result was obtained by Ghanbarzadeh et al. [20], who reported that the addition of CMC in all concentrations decreased the water solubility of starch films; however, the increase in the CMC contents, the moisture content increased. The water solubility of the edible film-based BC, which ranges from 30–60% based on this study and research by Atta et al. [24] and Wang et al. [14], is lower than starch-based edible films, which ranges between 80–90% [25]. The water solubility of edible film-based BC is low since cellulose is hydrophilic and insoluble in common solvents due to abundant free hydroxyl (OH) groups that form strong intra-and intermolecular hydrogen bonding between the chains [26].

Rangel-Maron et al. [27] also reported that the increase in glycerol concentration contributes to the moisture content of edible film due to the ability of glycerol to retain the water. Glycerol is the simplest glyceride compound with hydrophilic and hygroscopic hydroxyl, making it easy to bind with water [28]. Moisture mass transfer between packaged products and the environment should be limited by packaging films [28]. Because physicochemical and microbiological deterioration is greatly affected by moisture levels inside the package, the barrier capacity of a packaging film can extend the shelf life of a product [29,30].

### 3.3. Mechanical Properties of Edible Film

The mechanical properties of the edible film-based BC, including tensile strength (T), elongation at break (E), and Young’s modulus (Y), are presented in Table 3. The mechanical properties of the films are determined by the interactions between the components, namely the formation of solid molecular bonds between the chains or the formation of abundant bonds. The tensile strength indicates the maximum tension that the film can withstand, whereas the elongation at break is the maximum change in length of a test specimen subjected to tension before breaking, and this parameter aids in determining the flexibility and stretchability of films [31]. Young’s modulus indicated the material’s stiffness and described the film’s flexibility and mechanical properties related to the chemical structure [32].

Table 3 shows that adding CMC and glycerol up to 1% increased the tensile strength and Young’s modulus of the films, but if the concentration of CMC and glycerol was increased to 1.5%, it could decrease the tensile strength and Young’s modulus. On the other hand, elongation at break films increased with increasing concentrations of CMC and glycerol. These results indicated that the addition of CMC and glycerol to the BC could influence the mechanical properties of the film. The interaction of a mixture between BC, CMC, and glycerol as edible BC composite film components affects its mechanical properties.

Tensile strength and Young’s modulus of BC edible films increased in the presence of CMC and glycerol due to the increased interaction between the hydroxyl group of glycerol and the CMC in the BC matrix network during the mixing and drying of films [33]. Bipolar reactions and the effect of existing charges can also increase the tensile strength [34]. As the attractive force between the molecules making the edible film increases, the structural strength also increases [33]. Such condition is related to microfibrils and CMC contained by the BC structure, a cellulose derivative with hydroxyl groups forming intermolecular hydrogen bonds between carboxylic group in the CMC molecular backbone of edible films [33,34].

The higher glycerol concentration as plasticizer decreased the tensile strength and Young’s modulus but increased elongation at break (Table 3). The results are supported by the observations of Asl et al. [35], who stated that plasticizer could easily be inserted between polymer chains, resulting in a “cross-linker” effect that reduced the free volume and segmental mobility of the polymer, caused by decreasing the mechanical strength of the films and enhancing their flexibility, resulting in an increased elongation at break of films.

### 3.4. *Chemical* *Structure Characteristics of Edible Film Using FTIR*

**Figure 2:**
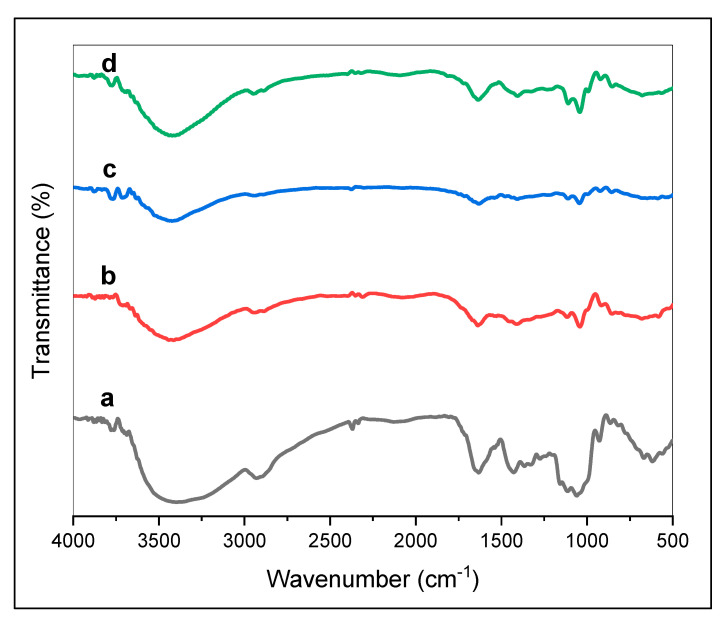
FTIR *edible film* spectrum using BC material from sago liquid waste. (**a**) EF_0-0_ (without CMC and glycerol), (**b**) EF_1-1_ (CMC 0.5%; glycerol 0.5%), (**c**) EF_2-2_ (CMC 1%; glycerol 1%), (**d**) EF_3-3_ (CMC 1.5%; glycerol 1.5%).

The chemical structure characteristics of edible films were analyzed using Fourier transformation infrared based on the functional groups making up the edible film. Analysis results of the FTIR edible film are shown in Figure 2.

Investigation of the chemical arrangement of the edible film BC bond was expressed through visualization of the FTIR spectrum (Figure 2). In order to analyze the influence of additives of CMC and glycerol on the chemical structure of BC film, an FTIR spectrum in the 500–4000 cm^−^^1^ range was examined. The characteristic bands of BC can be seen in all spectrums of BC composite films (Figure 2). The FTIR spectra of BC (Figure 2a) revealed distinct peaks at 3399 cm^−^^1^, 2929 cm^−^^1^, 1634 cm^−^^1^, and 1365 cm^−^^1^, and 1429 cm^−^^1^, corresponding to O−H stretching, C−H alkane stretching, and asymmetric CH_2_ stretching, O−H deformation, and CH_2_ deformation, respectively [6,24,36]. The -1,4 bond vibrations are characterized by an IR peak in the region of 850–860 cm^−^^1^ (Figure 2). The hydroxyl groups in cellulose help to form various types of inter-and intramolecular hydrogen bonds, and the formation of inter-and intramolecular hydrogen bonds in cellulose is essential for the physical properties of cellulosic materials [24]. The FTIR spectrum ranges between 3400–3410 cm^−^^1^ detected of edible BC composite films (Figure 2b–d). The peak at 3410 cm^−1^ originated from the stretching of –OH groups of cellulose and glycerol [24]. The absorbance peak at wavenumbers 3400–3435 cm^−^^1^ became slightly broad due to the –OH moiety interaction in the polymer backbone caused the increase of mechanical properties in the BC/CMC composite films [37]. In terms of structures, the band at 1637 cm^−1^ was due to water in the amorphous region. The presence of a strong absorption band at 1602 cm^−^^1^ is shown in Figure 2b–d; it confirms the presence of the CMC COO group [37].

### 3.5. *Surface Morphology of Edible BC Film Using SEM*

**Figure 3:**
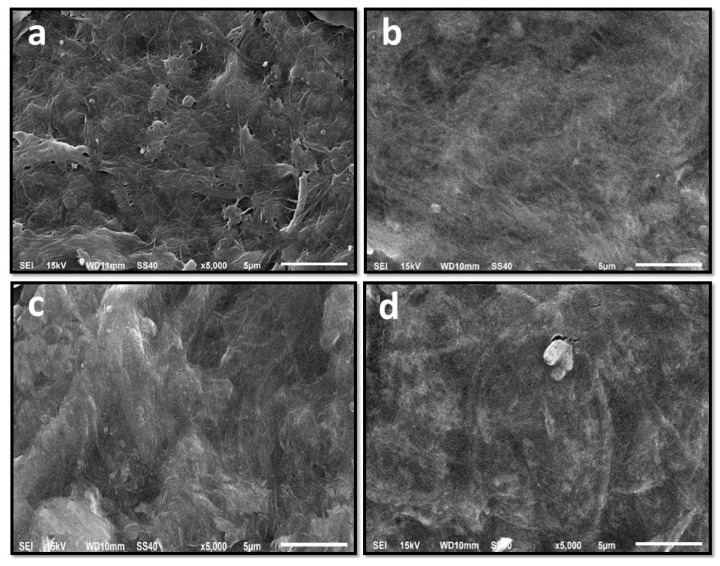
SEM of BC from sago liquid waste film. (**a**) EF_0-0_ (without CMC and glycerol), (**b**) EF_1-1_ (CMC 0.5%; glycerol 0.5%), (**c**) EF_2-2_ (CMC 1%; glycerol 1%), (**d**) EF_3-3_ (CMC 1.5%; glycerol 1.5%).

Scanning electron microscopy (SEM) analysis was carried out to determine the surface morphology characteristics of the edible film layer. The SEM micrographs of pristine BC from sago liquid waste film and BC-based composite films are shown in Figure 3. The pristine BC film (without CMC and glycerol) had a typical reticulated fibrous network structure with randomly distributed fibers (Figure 3a). In contrast, the SEM micrograph of the BC-CMC and glycerol composite film showed a high packing density of cellulose fibrils and exhibited a firm, smooth, and more homogeneous fibrous surface (Figure 3b–d). Increasing the concentration of CMC and glycerol makes the surface of the edible film smoother and has less pores than without CMC and glycerol (Figure 3b–d). This result indicates that incorporating glycerol and CMC reduced the surface roughness, which would affect the physicomechanical and biological features of the composite films [14]. The SEM results follow some previous studies, which show that the microstructural surface of BC-based composites with glycerol and CMC [24,38] and other materials show compact and homogenous surface morphologies.

### 3.6. Application of *Edible Film* as Food Packaging

**Figure 4:**
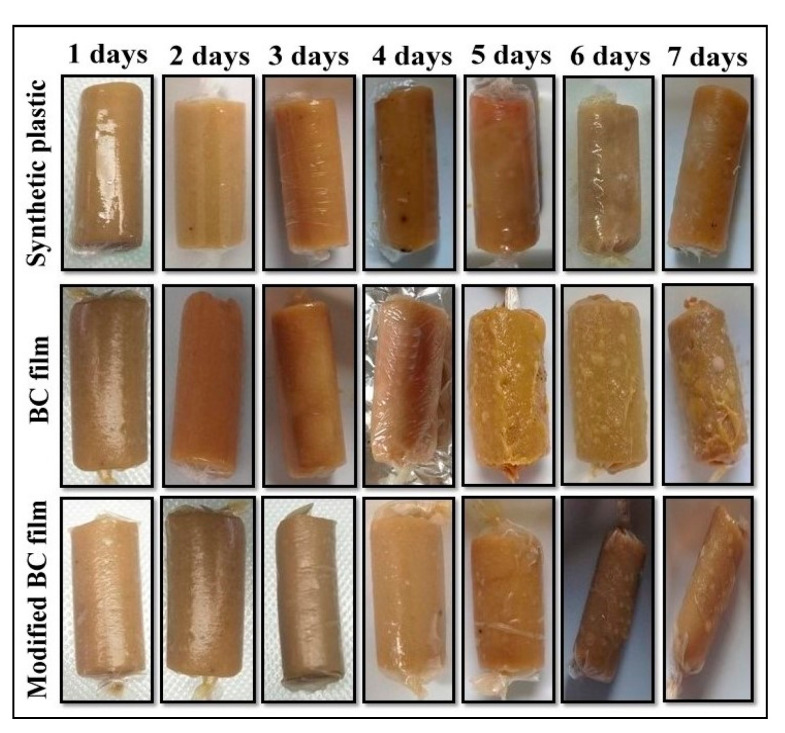
The photographs of meat sausage packaged by synthetic plastic (control), native BC film, and modified BC film were stored at room temperature.

The quality of food is affected by the length of the product storage time. Therefore, food packaging plays an important role in maintaining the quality and shelf life of food products. The use of edible film active packaging is an innovative way of extending shelf life and maintaining the quality of food products both physically, chemically, and biologically. On the fifth day, Organoleptic and physical observations of sausages packaged using synthetic plastic, modified BC film, and native BC film, included a mushier texture, a yellower color, and the appearance of white spots (Figure 4). Physical changes in sausages can be caused by microorganisms [39], chemicals, and other ingredients used as mixed ingredients for making sausages [40,41,42].

**Figure 5:**
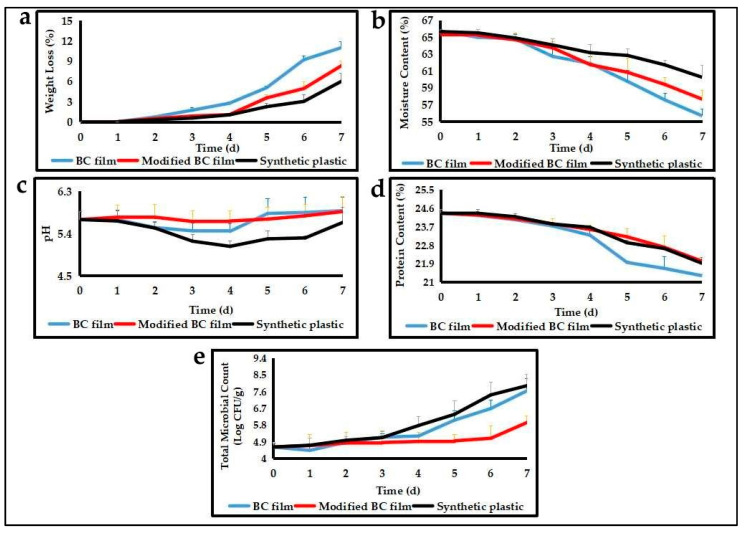
Evaluation of quality of meat sausage packaged by synthetic plastic (control), native BC film, and modified BC film stored at room temperature. (**a**) The weight loss, (**b**) moisture content, (**c**) pH value, (**d**) protein content, and (**e**) total microbial count.

The application of edible film as food packaging tested on meat sausage intended to determine the ability of edible film packaging to maintain the quality of packaged food. At ambient temperature, the edible film was applied in meat sausage for 7 days. Indicators of the quality of packaged meat sausages include weight loss, moisture content, pH value, protein content and total microbial count, which is shown in Figure 5.

Figure 5a shows that the percentage of weight loss of packaged meat sausages increased during storage, but on the contrary, the moisture content decrease (Figure 5b). These results indicate that packaged meat sausages evaporated, causing increased weight loss and decreased moisture content. Similar results were reported by Wulandari et al. [43] and Zamudio-Flores et al. [44], meaning that the weight loss of sausages covered with the film was related to moisture content. Ziegler et al. [45] also explained that, during the storage period, there was water loss due to evaporation and transpiration in the form of water vapor, resulting in a decrease in water content and an increase in the percentage of weight loss in food. Figure 5a also shows that the percentage of the weight loss of meat sausage packed using edible BC film and modified BC film was higher than synthetic plastic packaging. This means that edible film-based BC is still not good at inhibiting the occurrence of water evaporation, causing the meat sausage to decrease in water content. However, meat sausage packaged by modified BC (addition CMC and glycerol) film showed lower weight loss compared to native BC film (without CMC and glycerol) (Figure 5a). The result indicated that the addition of CMC and glycerol to the BC film could improve the function of the film to maintain water evaporation in packaged food products. The finding is supported by the moisture content (MC) of modified BC film, which is higher than moisture content BC film (Table 1). Janjarasskul and Krochta [46] stated that moisture content is used to analyze the barrier of water evaporating through the packaging materials. The presence of CMC as a surfactant in the edible film plays a role in reducing surface tension and superficial water activity, which can ultimately reduce the water loss of food packaged. This is confirmed by the research reported by Rodriguez et al. [47] that the addition of surfactants in the edible film formulation can significantly reduce the water evaporation rate.

Figure 5c shows that the pH value of meat sausage packaged by modified BC film tends to be constant during the storage period while, with sausage packaged by BC film and synthetic plastic (control), there was a change in pH value during storage. These results indicated that BC films modified with CMC and glycerol maintained a constant pH in meat sausages during storage. The ability of the modified BC films to keep the constant pH of the packaged food product indicates that the packaging can protect the quality of food products from damage caused by the growth of microorganisms. This is supported by Ludwicka et al. [5] who stated that pH change is a sign of adverse changes in foodstuff quality that can be correlated with the growth of microorganisms, and that the prominent role of pH detectors is to monitor the quality of packaged food. Ludwicka et al. [5] also stated that the addition of additives material, such as CMC and glycerol to BC, could improve the physicochemical properties of the film so the film can protect food from microorganisms contamination and against mechanical damage during the storage. 

The changes in pH of food packaged by BC films (native BC) and synthetic plastic (control) showed that the pH decreased from the third day to the fourth day and started to increase on the fifth day to the seventh day of storage (Figure 5c). This is probably due to the increased microbial growth in food packaged. The decrease in pH of meat sausage on third days is due to carbohydrate metabolism into lactic acid by lactic acid bacteria, and the fermentation results cause sausage pH to be lower [38,48]. In contrast, the increasing pH of meat sausage occurs because meat proteins undergo proteolysis into essential compounds, such as amino acids, indoles, and amines [48]. This result was supported by the decreased protein content in sausages during storage, especially on the fifth day (Figure 5d). The decrease in the protein content of packaged sausages indicates a proteolysis process that produces chemical compounds that are alkaline and causes the pH to increase [43].

Figure 5e showed that the number of microbes of meat sausages packaged by modified BC film tends to be constant until the sixth day of storage, while the number of microbes of sausage packaged by the native BC film and synthetic plastic increases on the fourth day. In this study, it was also known that the microbial growth in meat sausage packaged by synthetic plastic was higher than sausages packaged by edible BC film and modified BC film (Figure 5e). The results indicated that BC-based edible films can protect packaged food products from microbial contamination, and that edible film based on BC composite (modified BC film) is the best at protecting food from microbial contamination. A similar result was reported by Lan et al. [23], who reported that CMC composite films could inhibit bacterial growth. Ludwicka et al. [5] also reported that CMC composite films could protect packaged food from microbial contamination.

The ability of BC composite film packaging to protect packaged products from microbial contamination indicates that the packaging can maintain product quality. Lan et al. [23] stated that the composite film could improve food safety and quality. Microbial growth in packaged foods can cause physical and chemical changes, such as color, pH, and protein content causing decreasing food quality. This is supported by the results of this study which showed that sausages packaged by modified BC films with constant microbial counts (Figure 5e) tended to have no change in pH values during storage (Figure 5c). On the other hand, meat sausages packed with native BC films and synthetic plastics with microbial count increased during storage (Figure 5e), showing a change in the sausage pH value (Figure 5c). Ludwicka et al. [5] stated that the pH changes in packaged foods correlated with the growth of microorganisms, and these changes are a sign of decreased food quality. 

This study found that meat sausages packaged with synthetic plastic had the lowest percentage of weight loss (Figure 5a) and the highest moisture content (Figure 5b) compared to meat sausages packaged by BC film. This result indicates that the water content of sausages packaged by synthetic plastic is higher than sausages packaged by BC film, thus causing microbial growth in sausages packaged in synthetic plastics, which is also higher than sausages packaged by BC film (Figure 5e). These results are similar to those obtained by Chowdury et al. [49], who reported that the water content in food affects the weight of the food, and that water activity affects microbial growth in food. Martins et al. [50] also reported that the high water content in food will trigger microbial growth.

## 4. Conclusions

This study showed the potential of developing edible films based on BC produced by using sago liquid waste as a fermentation substrate. The mechanical properties of the edible film were affected by the addition of carboxymethyl cellulose (CMC) as stabilizers and glycerol as plasticizers in edible BC film products. The interaction of a mixture between BC, CMC, and glycerol as the components of edible BC composite film increased the thickness and the strength of the film, which then significantly increased the water solubility and decreased the moisture content. BC-based edible films from sago liquid waste can increase the storage life of meat sausage in microbiological terms. It elaborated that edible film-based BC could improve food safety through plastic packaging.

## Figures and Tables

**Table 1:** Edible film composition.

Treatment Code	Comparison of *edible film* Composition		
	CMC (%)	Glycerol (%)	Slurry Biocellulose (%)
EF_0-0_	0	0	100
EF_1-1_	0.5	0.5	99
EF_2-2_	1	1	98
EF_3-3_	1.5	1.5	97

**Table 2:** Thickness, water solubility (WS), and moisture content (MC) of the edible film with composition variation of CMC and glycerol.

Samples	Thickness (mm)	WS (%)	MC (%)
EF_0-0_	0.045 ± 0.006	30.81 ± 2.34	17.15 ± 0.82
EF_1-1_	0.065 ± 0.009	43.92 ± 2.80	19.30 ± 1.36
EF_2-2_	0.074 ± 0.004	42.54 ± 1.78	27.06 ± 0.64
EF_3-3_	0.083 ± 0.004	32.02 ± 1.10	33.72 ± 0.68

Results are expressed as mean ± standard deviation (n = 3) of the film-based BC prepared without CMC and glycerol (EF_0-0_), and with CMC and glycerol 0.5%–0.5% (EF_1-1_), 1%–1% (EF_2-2_), 1.5%–1.5% (EF_3-3_).

**Table 3:** Mechanical properties of edible BC film with composition variation of CMC and glycerol.

Treatment Code	Mechanical Properties		
	Tensile Strength (MPa)	Elongation at Break (%)	Young’s Modulus (GPa)
EF_0-0_	5.02 ± 0.70	10.27 ± 1.46	2.93 ± 0.44
EF_1-1_	9.30 ± 1.31	21.82 ± 1.08	4.49 ± 0.57
EF_2-2_	17.47 ± 1.17	25.60 ± 1.45	6.54 ± 0.12
EF_3-3_	7.67 ± 0.82	29.67 ± 1.04	5.78 ± 0.16

Results are expressed as mean ± standard deviation (*n* = 3) of the film-based BC prepared without CMC and glycerol (EF_0-0_), and with CMC and glycerol 0.5%–0.5% (EF_1-1_), 1%–1% (EF_2-2_), 1.5%–1.5% (EF_3-3_).

## Data Availability

Not applicable.
